# Born Too Soon: Accelerating actions for prevention and care of 15 million newborns born too soon

**DOI:** 10.1186/1742-4755-10-S1-S6

**Published:** 2013-11-15

**Authors:** Joy E Lawn, Mary V Kinney, José M Belizan, Elizabeth Mary Mason, Lori McDougall, Jim Larson, Eve Lackritz, Ingrid K Friberg, Christopher P Howson

**Affiliations:** 1 MARCH, London School Hygiene &Tropical Medicine, UK; 2 Saving Newborn Lives/Save the Children; 3 Saving Newborn Lives, Save the Children, Cape Town South Africa; 4 Institute for Clinical Effectiveness and Health Policy (IECS), Buenos Aires, Argentina; 5 World Health Organization, Geneva, Switzerland; 6 The Partnership for Maternal, Newborn and Children Health, Geneva, Switzerland; 7 Boston Consulting Group, Washington DC, USA; 8 Global Alliance to Prevent Prematurity and Stillbirth (GAPPS), Seattle, WA, USA; 9 Johns Hopkins University, Baltimore, Maryland, USA; 10 March of Dimes, White Plains, NY, USA

## Abstract

Preterm birth complication is the leading cause of neonatal death resulting in over one million deaths each year of the 15 million babies born preterm. To accelerate change, we provide an overview of the comprehensive strategy required, the tools available for context-specific health system implementation now, and the priorities for research and innovation. There is an urgent need for action on a dual track: (1) through strategic research to advance the prevention of preterm birth and (2) improved implementation and innovation for care of the premature neonate. We highlight evidence-based interventions along the continuum of care, noting gaps in coverage, quality, equity and implications for integration and scale up. Improved metrics are critical for both burden and tracking programmatic change. Linked to the United Nation's *Every Women Every Child *strategy, a target was set for 50% reduction in preterm deaths by 2025. Three analyses informed this target: historical change in high income countries, recent progress in best performing countries, and modelling of mortality reduction with high coverage of existing interventions. If universal coverage of selected interventions were to be achieved, then 84% or more than 921,000 preterm neonatal deaths could be prevented annually, with antenatal corticosteroids and Kangaroo Mother Care having the highest impact. Everyone has a role to play in reaching this target including government leaders, professionals, private sector, and of course families who are affected the most and whose voices have been critical for change in many of the countries with the most progress.

**Declaration:**

This article is part of a supplement jointly funded by Save the Children's Saving Newborn Lives programme through a grant from The Bill & Melinda Gates Foundation and March of Dimes Foundation and published in collaboration with the Partnership for Maternal, Newborn and Child Health and the World Health Organization (WHO). The original article was published in PDF format in the WHO Report "Born Too Soon: the global action report on preterm birth" (ISBN 978 92 4 150343 30), which involved collaboration from more than 50 organizations. The article has been reformatted for journal publication and has undergone peer review according to *Reproductive Health*'s standard process for supplements and may feature some variations in content when compared to the original report. This co-publication makes the article available to the community in a full-text format.

## Preterm birth as a marker for women's and children's health

The *Born Too Soon *report, published in 2012, drew global attention to the issue of preterm birth and reported that more than 1 in 10 of the world's babies are born too soon each year, 15 million each year [1]. As part of a series entitled "Born Too Soon" drawing from the report, this final paper summarises the problem, underlining the need for concerted action on both the prevention of preterm birth and care of the premature baby, to ensure every mother and every baby survives [2-6]. We then highlight evidence-based interventions for preterm birth in the context of the wider health system drawing on the other papers in this supplement, and here we focus on the implications for integrating and scaling up those available interventions in low- and middle-income countries where the coverage of care is lowest and the potential lives saved as a result. We also consider research gaps since advancing the research agenda is a critical need to reduce the global burden of preterm birth, requiring innovations for both prevention and care. Finally we detail the analyses for a mortality reduction target for preterm specific neonatal deaths and outline the specific roles all actors must play in this global effort to reduce preterm birth and care for premature babies, which is a marker of the health and care of women and girls, as well as of progress for child survival and development.

The actions identified aim to support the goals of the *Global Strategy for Women's and Children's Health *launched by the United Nations (UN) Secretary-General Ban Ki-moon in September 2010 to further advance progress for the Millennium Development Goal 4 for child survival and the *Every Woman Every Child *movement to mobilise action and resources for these goals [7]. By pooling our efforts with each organisation playing to its strengths, our shared goal, as epitomised in *Every Woman Every Child*, can be realised -- a day when pregnancies are wanted and safe, women survive, babies everywhere get a healthy start in life, and children thrive.

## Accelerating evidence-based action for prevention and care

**Figure 1:**
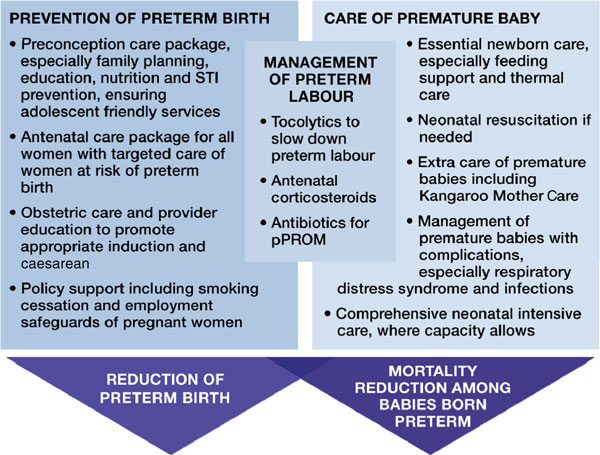
**Approaches to prevent preterm birth and reduce deaths among premature babies**. Source: Born Too Soon, Chapter 6 [75]. Dean et al., 2013 [4]; Requejo et al., 2013 [5]; Lawn et al., 2013 [6].

Addressing the burden of preterm birth has a dual track--prevention and care (Figure 1). Reducing risks before, during, and between pregnancies through preconception and antenatal care packages may help preterm birth prevention [4,5]. Actions taken during labour and birth, and particularly improved care of the neonate have been shown to have major impact [5,6]. For example, antenatal corticosteroids administered to a pregnant woman in preterm labour can prevent respiratory distress syndrome in premature babies reducing newborn mortality and morbidity [5]. In addition, many of these interventions, such as obstetric care and antibiotics for prelabour premature rupture of membranes (pPROM), also benefit maternal health and prevent stillbirths [8]. Interventions that have been identified through global reviews of the evidence are summarised elsewhere [4-6] and shown in Figure 1.

## Prevention of preterm birth is primarily a knowledge gap

Despite the burden of preterm birth, few effective prevention strategies are available for clinicians, policy-makers and program managers. Multiple studies in high-income contexts have attempted to prevent preterm birth, but have not yet identified high-impact interventions in the preconception and antenatal periods. Many interventions have been evaluated, and some have been identified as beneficial though limited in public health impact, such as therapy with progestational agents, which have only been studied in certain high-risk populations. Preliminary studies of interventions to reduce rates of elective caesarean births or inductions without medical indication before the recommended 39 completed weeks of gestation suggest an impact on prevention of early term deliveries in some high- and middle-income countries [9,10]. A recent study published in *The Lancet *examined preterm birth prevention potential in 39 high-income countries and estimated that if five interventions reached high coverage there would only be a 5% relative reduction of preterm birth rate from 9·59% to 9·07% of livebirths by 2015, averting an estimated 58,000 preterm births and saving US$3 billion annually [11]. These five interventions were: smoking cessation (0·01 rate reduction), decreasing multiple embryo transfers during assisted reproductive technologies (0·06), cervical cerclage (0·15), use of progesterone agents (0·01), and reduction of elective labour induction or caesarean delivery without medical indication (0·29). The limited number and effectiveness of available interventions for preterm prevention further underscores this critical major knowledge gap, and makes the case for a strategic and coordinated research effort to advance understanding of causes and mechanisms of preterm birth and identification of innovative solutions.

However, the low- and middle-income countries with the highest burden of preterm births also carry the greatest burden of higher-risk conditions for preterm birth that are preventable or treatable. Interventions such as family planning; prevention and management of sexually transmitted infections (STIs); use of insecticide-treated bednets and intermittent preventive treatment for malaria; identification and treatment of pre-eclampsia, and reduction of physical workload are examples of strategies that could improve birth outcomes in in low- and middle-income settings. Unfortunately, to date, few studies have assessed the impact of these interventions on preterm birth in these countries, particularly with accurate measures of gestational age [12]. The greatest potential for the global prevention of preterm birth, therefore, lies in a comprehensive, strategic, and sufficiently-funded research agenda of the causes of preterm birth and novel strategies for prevention [13]. This should be vigorously pursued.

There are some significant intrapartum interventions that reduce the impact of preterm birth. Antenatal corticosteroid injections given to women in preterm labour are highly effective at preventing respiratory distress syndrome in premature babies and associated mortality and long-term impairment, but remain under-used in many low- and some middle-income countries. There is, thus, a need for delivery research that can help understand context-specific reasons for the continued low coverage in these countries and identify ways to adapt known effective strategies for use in low-resource settings [14]. Tocolytic medicines rarely stop preterm labour, but may help delay labour for hours or days, allowing the baby additional precious time to develop before birth. Of course, any strategies to prolong labour, including delaying caesarean birth, must be evaluated against the potential risk of continued exposure of woman and foetus to sub-optimal conditions that may result in harmful effects. Further research is needed on short and long-term health consequences for mother and baby from efforts to prevent preterm labour [5].

## Care of premature babies is primarily an action gap

As evidenced by the large survival gap between babies born in high-income countries and those born in low- and middle-income countries, effective interventions exist to reduce death and disability in premature babies, yet this care does not reach the poor and most disadvantaged populations where the burden is highest [6]. There is a "know-do gap", or a gap between what is known to work and what is done in practice. Bridging this gap will be critical for saving premature babies globally, and must be linked to implementation research and context specific adaptation and innovation.

More than 60% of all premature babies are born in South Asia and sub-Saharan Africa which have the highest preterm birth rates [15] and half of births are currently in facilities. Most preterm births occur over 32 weeks of gestation (84%), and deaths in these babies can almost all be prevented and in most cases, intensive care is not needed [6] (Figure 1). It is possible to implement some evidence-based interventions for the care of premature babies at the community level through behaviour change initiatives and women's groups [16], as well as home-visit packages with extra care for premature babies, particularly breastfeeding support and awareness of the importance of seeking care when danger signs occur [17]. In a few countries, case management of neonatal sepsis is being scaled up using community-based health workers [18]. However, the highest impact interventions, notably access to quality intrapartum care and emergency obstetric and newborn care [19], require facility-based services. Antenatal corticosteroids and Kangaroo Mother Care (KMC) are evidence-based interventions that are feasible to scale up in low-resource settings and may serve as entry points for strengthening health systems [20,21].

## Scaling up preterm birth interventions within the existing health system

**Figure 2:**
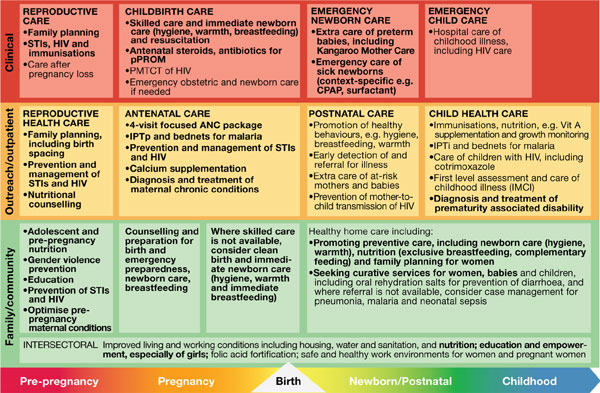
**Integrated service delivery packages for maternal, newborn and child health**. Source: Born Too Soon, Chapter 6 [75]. Adapted from (Kerber et al., 2007; Lawn et al., 2012; PMNCH 2012) [22,23,51]. Note: interventions for preterm birth are bold. Acryomns used: ANC = Antenatal care; CPAP = Continuous positive airway pressure; HIV = Human Immunodeficiency Virus; IMCI = Integrated Management of Childhood Illnesses; IPTp = Intermittent presumptive treatment during pregnancy for malaria; pPROM = prelabour premature rupture of membranes; STI = Sexually Transmitted Illness.

There is increasing global consensus around essential reproductive, maternal, newborn and child health (RMNCH) interventions [22,23], including those to address preterm birth (Figure 2). The goal is to achieve universal, equitable coverage and high quality in all these RMNCH interventions. Newborn babies, and especially premature newborns, are the most sensitive test of health systems function as these babies can die within minutes without the right care. For sustainable effect, interventions to prevent preterm birth in the preconception and antenatal periods and to reduce death and disability in premature babies must be integrated within the existing health system.

The continuum of care is a core organising principle for health systems emphasising linkages between healthcare packages across time and through various service delivery strategies [2]. An effective continuum of care addresses the health needs of the adolescent, woman, mother, newborn and child throughout the life cycle, wherever care is provided, whether it be at the home, primary care level or district and regional hospitals. Integrated service delivery packages of evidence-based interventions within the continuum of care have many advantages: cost-effectiveness is enhanced; available human resources are maximised; and services are more family-friendly, reducing the need for multiple visits [24]. Most importantly, they can help prevent stillbirths, improve prevention and care of premature babies and avert death and disability in women, newborns and children [25-27].

Interventions with the highest impact on the prevention of preterm birth and care of the premature baby in high-mortality and lower-resource settings can be integrated into these health service delivery packages, which exist in most health systems and involve links with maternal and child health services, as well as immunisation, malaria, HIV/AIDS, nutrition, family planning, and other related programs [22]. A schematic matrix of the basic health packages (Figure 2) outlines these packages spanning the continuum of care and through various service delivery modes within the health system, highlighting the interventions included to address preterm birth. The interventions within each package are based on multiple systematic reviews and are consistent with the Partnership for Maternal, Newborn and Child Health Essential Interventions report [23].

While these packages may exist in nearly all health systems, lower-income countries cannot scale up and implement all the individual RMNCH interventions within all the packages at once [25]. Packages usually are initially comprised of the essential interventions and then increase in complexity over time according to local needs and capacity. For example, the Antenatal Care package may start as the WHO focused four visit package and then later add on diabetes screening and routine ultra-sound as the system capacity and funding increases [26]. The functionality of health systems, such as human resource capacity, health facility infrastructure, supply and demand systems, financial resources, government stewardship, district-level management and use of data, will also determine the coverage, quality and rate of change within the continuum of care [28].

## Closing gaps in coverage, equity and quality

In order for health services to save the maximum number of lives, coverage, quality and equity need to be high; thus ensuring high coverage of care means reaching every woman, mother-to-be, mother, newborn, child and family with targeted interventions. Providing quality care involves doing the right thing at the right time. Providing equitable care means ensuring care for all according to need, rather than income, gender or other social grouping. This holds true for the existing inequalities in care within and across high-income as well as low- and middle-income countries. Previous papers in this supplement have identified gaps in coverage, quality, equity and metrics for care during preconception, pregnancy and care of preterm newborns [4-6].

**Figure 3:**
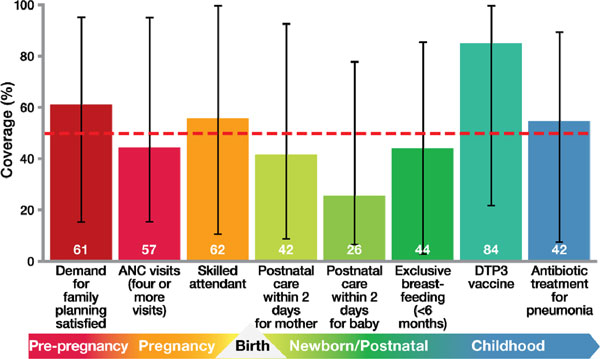
**Coverage along the continuum of care for 75 Countdown to 2015 priority countries**. Source: Countdown to 2015 (Requejo et al., 2013) [29]. Note: Eight selected Commission on Information and Accountability for Women's and Children's Health indicators, showing median for Countdown priority countries. Acryomns used: ANC = Antenatal care; DTP3 = Three doses of diphtheria, tetanus and pertussis vaccine.

Current coverage levels for eight indicators across the continuum of care, chosen by the United Nations Commission on Information and Accountability for Women's and Children's Health, are tracked for the 75 priority Countdown to 2015 countries which collectively account for 90% of maternal, newborn and child deaths [29]. Currently, essential care reaches only half of the people in need (Figure 3), and there is a wide variation in coverage levels among countries, with some countries achieving nearly universal coverage and others reaching less than a quarter of the population. Demand for family planning satisfied and antenatal care coverage, even though feasible through primary care services, still leave out many women, especially the poorest. In addition, quality gaps are a missed opportunity for reaching families; for example, when a midwife is present at birth but is not equipped to prevent post partum haemorrhage or to resuscitate a baby who does not breathe [5,6]. Substantial progress is still needed for the reduction of maternal and newborn deaths, especially for effective, high quality at the vital contact times (e.g., skilled attendant at birth and postnatal care) [29]. Currently, there are no routine data available for many of the interventions for preterm birth prevention and care.

## A research pipeline to address preterm birth

Greater investment in research and, in particular, into discovery of the many complex and interrelated factors causing preterm birth is needed to strengthen prevention and offers a potential over the longer term for significant reductions in mortality, childhood disability and healthcare expenditure. For care of preterm babies, the emphasis is on rapidly scaling up implementations, so that the maximum number of preterm babies and their mothers benefit. Implementation research is needed to understand the most efficient means of scaling up evidenced based solutions. In this way, hundreds of thousands of lives could be saved with the application of current knowledge.

Preterm birth is not a single condition, but a single outcome (birth before 37 completed weeks) due to multiple causes. Hence, there will not be a single solution, but rather an array of solutions that address the various biological, clinical, behavioural and social risk factors that result in preterm birth. This supplement identifies risks for preterm birth and the solutions needed to reduce those risks across the RMNCH continuum; yet for many of these risks, we do not have effective solutions. Important research priorities have been highlighted [4-6]. A strategic research approach is needed to understand why babies are born preterm or as stillbirths, how to identify women at risk, to test strategies for prevention and care, and reduce death and disability rates for preterm neonates.

Important research themes can be summarised across the research pipeline of description, discovery, development and delivery science, showing the dual agenda of preventing preterm birth and addressing the care and survival gap for babies born preterm (Table 1) [30]. For the preterm prevention research agenda, the greatest emphasis is on discovery and descriptive research, which is a longer-term investment. For the premature baby care agenda, the greatest emphasis is on development and delivery research, with a shorter timeline to impact at scale.

**Table 1:** A research pipeline advancing knowledge to address preterm birth.

	Description	Discovery	Development	Delivery
	Characterise the problem	Understand the problem	Create and develop new interventions	Advance equitable access to interventions
Research aim	Descriptive epidemiology to understand determinants, advance definitions	Development of new interventions or adapting or improving existing interventions	Development of new interventions or adapting or improving existing interventions	Delivery of interventions at scale through innovative approaches

Preterm prevention research themes	• Improve collection, analysis, interpretation, application of epidemiological data for: • Refining, disseminating standard definitions of exposures, outcomes and phenotypes • Further understanding of risk factors for preterm birth • Monitoring and evaluating impact of interventions • Improving the estimates and data collection systems	• Increase knowledge of the biology of normal and abnormal pregnancy • Better understand modifiable mechanisms contributing to preterm birth (e.g., preconceptual or antenatal nutrition, infection and immune response) • Advance understanding of underlying pathophysiology of preterm newborns and impact of co-morbidities on outcomes in different country settings	• Create, develop new interventions (e.g., novel approaches to preventing preterm birth) • Adapt existing interventions to increase effect, reduce cost, or expand utilisation and access	• Evaluate impact, cost and process of known interventions to reduce preterm birth (e.g., family planning, STI management, malaria prevention) • Social behaviour change research to address lifestyle factors and other risks for preterm birth • Effective approaches to increase use of antenatal steroids in low- and middle-income settings
			
Premature baby care research themes			• Create new devices and drugs for preterm babies that are feasible to use in low-income settings • Adapt existing interventions to increase effect, reduce cost, and/or improve deliverability in challenging settings or at community level (e.g., robust, simpler technologies)	• Implementation research to adapt and scale up context-specific packages of care for preterm babies (e.g., examining task shifting, innovative commodities, etc.) • Create and implement effective community-based approaches (e.g., community health workers home visit packages, women's' groups)

Typical timeline to impact	Near-term to Long-term (2 to 15 years)	Long-term (5 to 15 years)	Medium-term (5 to 10 years)	Near-term (2 to 5 years)

Source: Born Too Soon, Chapter 6 [75]. Adapted from Lawn et al., 2008; Rubens et al., 2010.

### Descriptive research

Improved and consistently applied epidemiologic definitions and methods, with clearly defined preterm phenotypes, are the foundation for improved understanding of the burden of preterm birth [31,32] and addressing the multiple and often interrelated causes of preterm birth. Simpler and lower-cost methods for measuring gestational age are particularly needed in low- and middle-income countries where the burden of preterm birth is highest. Social and racial disparities in preterm birth rates are a major issue, yet remain poorly understood. Another important need is for standardised methods for diagnosing and treating prematurity-related impairment in childhood and more consistent measures and timing for assessing multi-domain impairments [33,34].

### Discovery research

Discovery research focuses on better understanding the causes and mechanisms of preterm birth and elucidating factors that regulate uterine quiescence, initiation of labour, and the multiple host, agent, and environmental factors that cause aberrations in these normal processes of pregnancy. Understanding the reasons for racial and ethnic disparities in preterm birth will advance the field of pregnancy health broadly, as well as accelerate solutions for those populations most in need. A multi-disciplinary approach is needed to identify women at risk and discover new strategies for prevention including potential biomarkers, such as genomic, microbial, immunologic, and hormonal factors.

Although infectious and inflammatory processes contribute to a high proportion of early spontaneous preterm births [35], antibiotic treatment of reproductive tract infections, especially bacterial vaginosis and other remote site infections, has generally failed to reduce preterm risk [36]. Many pre-existing chronic conditions and medical complications of pregnancy may result in increased risk of preterm delivery, such as pre-eclampsia, hypertension, aberrations in placentation and placental growth, diabetes and infectious diseases. Identifying mechanisms of these conditions, and strategies for early detection, prevention, and care, represent an important need for reducing the global burden of preterm birth. New strategies for prevention are particularly urgent for use in low- and middle-income settings where rates are highest.

### Development research

Equipment and commodities are considered essential for neonatal care units in high-income countries, yet for many such units in low-income settings, basic equipment and essential medicines are not available or functional. Development of robust, fit-for-purpose equipment, is a critical next frontier for referral care for premature babies in the settings where most die, especially for care in hospitals [6]. Some examples include technologies for ventilatory support, novel surfactant formulations, safe and effective intravenous fluid and drug administration, devices for testing bilirubin levels for jaundice and innovative phototherapy equipment [6]. New and effective methods for monitoring and management of maternal complications and preterm labour could make a major contribution. Commodities, such as antenatal corticosteroids, could reach more women and babies though innovation for example in single-dose syringes or, ideally, needle-free devices [37].

### Delivery research

Delivery or implementation research addresses how interventions can be best implemented, especially in resource-constrained settings where coverage inequalities are more pronounced so that all families are reached with effective care. Implementation research and program evaluation evaluates how best to achieve wide scale coverage of interventions, including prevention particularly family planning and such as care of women with infectious diseases such as malaria, HIV and STIs; improved nutrition; smoking cessation; and reducing maternal workload. In many high-income countries and those with emerging economies, there is evidence of an increase in late preterm deliveries due to elective inductions and caesareans without clear medical indication [38]. More information is urgently needed from both providers and patients on the reasons for these shifts in clinical practice and how to promote more conservative obstetric management.

The vast majority of published studies on neonatal care relate to high-technology care in high-income settings [39]. Implementation research from low- and middle-income settings is critical to inform and accelerate the scale up of high-impact care, such as KMC and neonatal resuscitation [19,21,40]. Evaluation of context-specific neonatal care packages regarding outcome, cost and economic results is important, including adaptations such as task shifting to various cadres and use of innovative technologies [41]. There is also a need to understand how to screen more effectively for and treat possible prematurity-related cognitive, motor and behavioural disabilities, including in older children. In addition, the economics of preterm birth prevention and care, including the cost-benefit and cost-effectiveness of interventions delivered singly or as a package across the continuum of care and in different settings and populations as well as the costs of doing nothing, need to be better studied [12,42].

### Building the platform to accelerate research

Underlying this entire research agenda is the development and implementation of the capacity to advance the science of prevention of preterm birth, manage preterm labour and improve care of premature babies. Standard case definitions of the types and causes of preterm birth are being developed [31,32] and will be critical to accelerating discovery and making comparisons across studies from basic science to clinical trials and program evaluation. Multi-country studies in middle- and low-income countries tracking pregnant women with improved and accurate gestational dating may help contribute to improved pregnancy monitoring and a better understanding of all pregnancy outcomes for women, stillbirths and newborns. Improved communication and collaboration among researchers investigating these linked outcomes will provide an opportunity to accelerate the discovery, development and delivery of innovation, especially across disciplines and between laboratory benches and remote and under-resourced hospitals. Expanding training, research opportunities and mentorship for researchers in low-income settings hold great promise in developing a pipeline of expertise to advance the science with the skills to use this science effectively to promote change [30].

## Potential for lives saved

**Figure 4:**
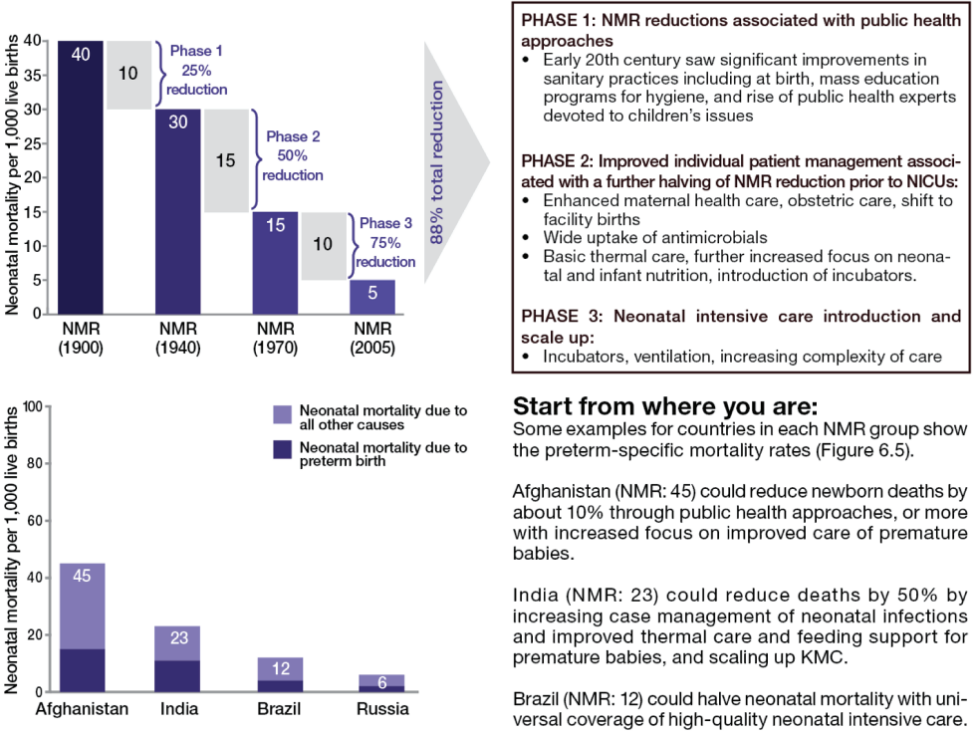
**Historical phasing of reductions in neonatal mortality rates in the United Kingdom and United States during the 20th century**. Source: Born Too Soon, Chapter 6 [75]. Data sources for UK and US historical data: (CDC, 2012, Office for National Statistics, 2012, NIH, 1985, Smith et al., 1983, Jamison et al., 2006, Lissauer and Fanaroff, 2006, Baker, 2000, Philip, 2005, Wegman, 2001) [54-62]. With thanks to Boston Consulting Group. Note: more information on history of neonatal mortality reduction in UK and USA available (Lawn et al, 2013) [6]. Data sources for Afghanistan, India, Brazil, and Russia from Child Health Epidemiology Reference Group/World Health Organization cause of death estimates for 2010 from Liu et al., 2012 [50].

**Figure 5:**
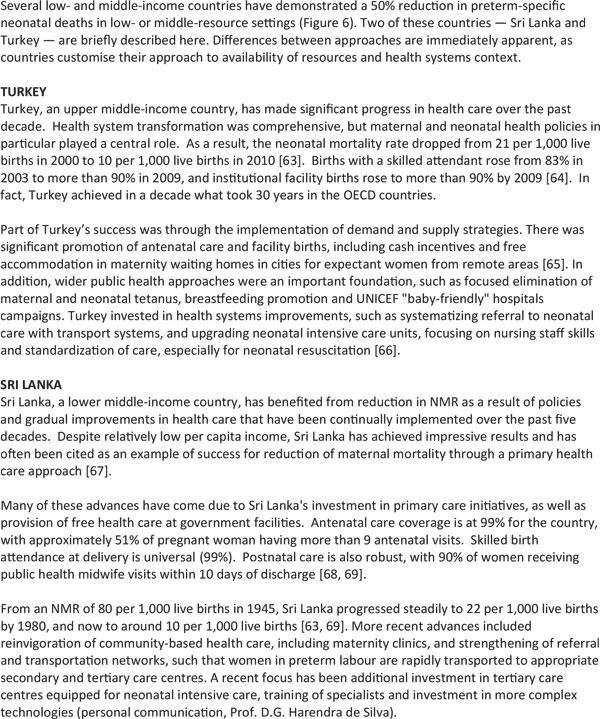
**Countries that have halved their deaths due to preterm birth in just one decade**.

**Figure 6:**
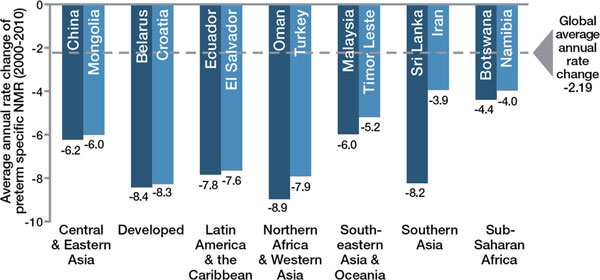
**Well-preforming countries for preterm-specific neonatal mortality reduction by region**. Source: Born Too Soon, Chapter 6 [75]. Analysis conducted using data from Liu et al., 2012 [50]. Credit: Boston Consulting Group with the Global Preterm Birth Mortality Reduction Analysis Group.

To understand the impact of evidence-based interventions on deaths due to complications of preterm birth, we considered analyses including historical data from high income countries (Figure 4), recent change in middle income countries (Figures 5 and 6) and a new analysis using lives saved modelling.

### History lessons from neonatal mortality reduction in high income countries

The historical data from the United States and United Kingdom (Figure 4) shows that a moderate increase in coverage of selected interventions results in a mortality reduction, even in the absence of neonatal intensive care. A number of lessons can be drawn from this historical data:

Basic care and infection case management interventions have an effect on neonatal deaths and on deaths amongst moderate and late preterm births, which account for over 80% of preterm births.

More targeted care is necessary for reducing deaths among babies 28 to <32 weeks and this reduction could be accelerated as higher-impact interventions are now known, such as antenatal corticosteroids, surfactant, KMC and other enhanced methods of infant warming and feeding which were not available in the mid-20th century in the United States and United Kingdom.

Intensive care may be necessary to reduce deaths among extremely premature babies (< 28 weeks), who account for 5% of all premature babies though a larger proportion of deaths.

### Lives saved modelling for preterm mortality reduction

A Lives Saved Tool (*LiST*) analysis examining projected lives saved with interventions for preterm birth was conducted for 75 Countdown to 2015 priority countries (These countries are: Afghanistan, Angola, Azerbaijan, Bangladesh, Benin, Bolivia, Botswana, Brazil, Burkina Faso, Burundi, Cambodia, Cameroon, Central African Republic, Chad, China, Comoros, Congo, Democratic Republic of the Congo, Côte d'Ivoire, Djibouti, Egypt, Equatorial Guinea, Eritrea, Ethiopia, Gabon, The Gambia, Ghana, Guatemala, Guinea, Guinea-Bissau, Haiti, India, Indonesia, Iraq, Kenya, Democratic Republic of Korea, Kyrgyz Republic, Lao People's Democratic Republic, Lesotho, Liberia, Madagascar, Malawi, Mali, Mauritania, Mexico, Morocco, Mozambique, Myanmar, Nepal, Niger, Nigeria, Pakistan, Papua New Guinea, Peru, Philippines, Rwanda, Sao Tome and Principe, Senegal, Sierra Leone, Solomon Islands, Somalia, South Africa, South Sudan, Sudan, Swaziland, Tajikistan, United Republic of Tanzania, Togo, Turkmenistan, Uganda, Uzbekistan, Vietnam, Yemen, Zambia, and Zimbabwe.). *LiST *is a free and widely used module in a demographic software package called Spectrum, which allows the user to compare the effects of different interventions on the numbers of maternal, neonatal and child deaths and stillbirths, as well as stunting and wasting [43]. The modelling methods have been widely published including discussions of the limitations, which are particularly related to the lack of coverage data for many of the specific interventions [44-47].

Table 2 shows the interventions included in the *LiST *analysis that prevent preterm births and improve survival of premature babies. We considered the period from 2010 to 2015 and then through 2025 to allow for a more feasible time frame to scale up care and progress on the prevention agenda. The results of the *LiST *analysis found that 84% of premature babies (more than 921,000 lives) could be saved in 2025 if these interventions were made universally available (95%). Full coverage of antenatal corticosteroids alone resulted in high mortality reductions, a 41% decrease from 2010 [20]. Implementing KMC alone also suggests that a high reduction of deaths could be achieved [21], averting approximately 531,000 neonatal deaths in 2025. If these two interventions were added to existing health system packages, especially noting the recent shifts to more facility births in Africa and Asia, then a high impact is possible even in a relatively short time frame.

**Table 2:** Estimated lives saved of premature babies in settings with universal coverage of interventions

Intervention reaching 95% coverage	Also saves mothers or other babies	By 2015		By 2025	
			
		% Deaths averted	Lives saved	% Deaths averted	Lives saved
Family planning*	M, SB, N	24	228,000	32	345,000
Antenatal corticosteroids	N	40	373,000	41	444,000
Antibiotics for pPRoM	N	9	85,000	9	101,000
Immediate assessment and simple care of all babies	N	5	44,000	5	53,000
Neonatal resuscitation	N	7	65,000	7	77,000
Thermal care	N	15	142,000	16	171,000
Kangaroo mother care	N	48	452,000	48	531,000
Interventions implemented together	M, SB, N	81	757,000	84	921,000

Note: interventions marked with M will also save maternal lives, SB would avert stillbirth, and N will save newborns dying from causes other than preterm birth.

* Family planning scaled to 60% coverage or to a level whereby the total fertility rate is 2.5. Note that obstetric care would also have an impact, but is not estimated separately

## Targets for action by 2025

**Figure 7:**
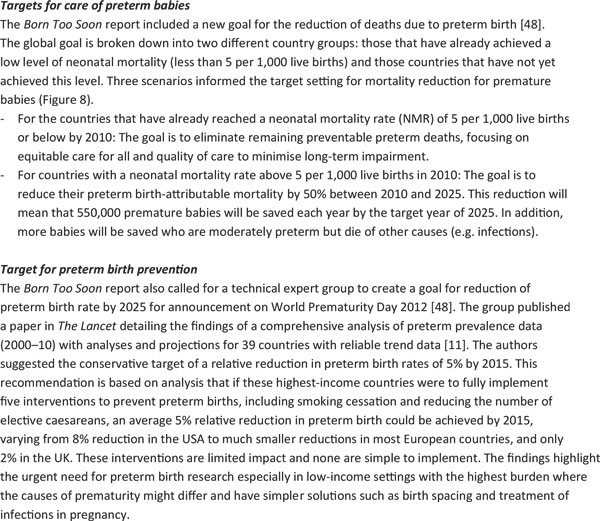
**Targets for action by 2025**.

The *Born Too Soon *report initiated a process towards achieving goals for preterm birth prevention and presented a new goal for the reduction of deaths due to complications of preterm birth (Figure 7) [48]. The latter goal was set through consultation by a group of technical experts, and several analyses were undertaken to inform this target, notably (1) projections by country of the deaths due to preterm birth from now until 2025, assuming no change in trends and assuming expected changes in Gross National Income (GNI); (2) reduction in preterm-specific neonatal mortality if the historical trends from the United Kingdom or the United States (Figure 4) were applied or if more rapid recent reductions in middle-income countries were applied (Figures 5 and 6); (3) preterm-specific neonatal mortality reductions predicted based on coverage changes according to the Lives Saved Tool Modelling (Table 2). These analyses used data from UN demographic projections of births [49] and the Child Health Epidemiology Reference Group/World Health Organization neonatal cause of death time series, 2000 to 2010 [50].

**Figure 8:**
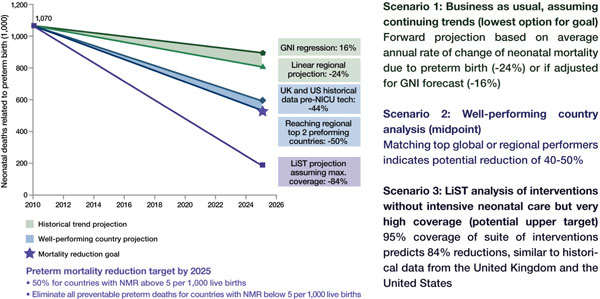
**Results of three scenarios of preterm-specific mortality reduction to 2025**. Source: Born Too Soon, Chapter 6 [75]. Analysis conducted by Mortality Reduction Goal Group and Boston Consulting Group using multiple data sources (Liu et al., 2012; EIU GDP projections 2010 to 2030; World Population Prospects, 2010; UN Department of Economic and Social Affairs; LiST analysis) [50,52,53]. Note: Analysis is for countries with NMR of more than 5 per 1,000 live births; other countries are excluded. Interventions in the LiST analysis included KMC, antenatal corticosteroids, antibiotics for pPRoM, skilled birth attendance, and others.

Using the results from analyses of the three future scenarios (Figure 8), a target for mortality reduction of preterm births was set and agreed by the technical experts (Figure 7).

### Scenario 1: "Business as usual"

Should governments and the global community take no further direct action to address deaths due to preterm birth, mortality will decline by 24% by 2025 according to an analysis of regional trends over the past decade and forward projection (or 16%, if the projection is based on forecasted GNI change) (Figure 4). Given this scenario and taking into account changing numbers of births, the global total of preterm deaths will not reduce significantly by 2025, with around 900,000 premature babies continuing to die every year.

### Scenario 2: Countries take action to catch up with top performers within their region

Preterm mortality could be halved by 2025 if governments took action now to match the top performers within their regions or to match the historical reductions in the United States and the United Kingdom from basic interventions before widespread use of intensive care (Figure 4). The examples of Sri Lanka and Turkey (see Figure 5) present examples of significant reduction in mortality, halving deaths in 10 years linked to scale up of intensive care. Even those countries with higher mortality rates that are not yet ready to scale up intensive care could see a 50% reduction as shown in the mid-20th century in the United States and the United Kingdom. This reduction is achievable with improved essential care of premature babies and better case management of infections and respiratory distress syndrome, especially since the deaths of moderately-preterm babies are the most common and preventable ones.

There are high-impact, cost-effective interventions currently at low coverage [5,6], such as antenatal corticosteroids and KMC, that could significantly accelerate progress, which were not available in the United States and the United Kingdom in the middle of the 20th century when the neonatal mortality rate (NMR) was significantly reduced. Hence, it would be expected, with the inclusion of these and other innovations, that mortality reduction could be more rapid than for the historical examples.

### Scenario 3: Countries achieve universal coverage of basic interventions

Should governments adopt universal coverage of interventions (95%) ensuring that every woman and child who needs an intervention receives it, then, according to the *LiST *analysis (Table 2) and the historical data (Figure 4), countries could achieve an 84% reduction of 1.1 million deaths due to preterm birth complications. While ensuring a 95% coverage rate is ideal and would result in a major mortality reduction, this process will take time. Initiating these changes can start to move countries toward their goal of preterm mortality reduction while also preventing death due to other causes of newborn death, as well as maternal deaths and stillbirths, through shared interventions such as skilled care at birth.

### Call to action

*Born Too Soon *is sobering in the news of a large burden and in the personal stories of loss behind that burden. Yet this is also a story of hope in the significant opportunities for change, especially as we approach the final sprint for the MDG 4 target and aim to maintain momentum beyond 2015. These first-ever country estimates of preterm birth leave us without the excuse of ignorance [3]. In 2010, 15 million babies -- more than 1 in 10 births -- were born too soon, an emotional and economic toll on families, communities and countries. The problem is increasing - for the countries with 20-year trend data, the majority show an increase in preterm birth rates [3]. Additionally, the burden is not shared equally, with the impact of preterm birth falling most severely on the poorest families and in low- and middle-income countries where health systems are less prepared to respond. There are also high preterm birth rates in many high-income countries, including the United States. Preterm birth is a problem that we all share; therefore, the solutions must also be shared, and won through cooperation, collaboration and coordination of the many constituencies and stakeholders.

A number of specific actions, pursued by all partners and applied across the RMNCH continuum of care, will help prevent preterm birth and associated mortality, and have an immediate, profound and sustained impact on human capital. The seven constituencies, as identified by *Every Woman Every Child *[7], have four action themes, which link closely to the principles of Act, Monitor and Review recommended by the Commission on Information and Accountability for Women's and Children's Health.

#### Invest

Bring both financial and other resources to address maternal and newborn health and the burden of preterm birth.

#### Implement

• Adapt integrated packages of care, considering contexts, and tailored to local health service delivery channels.

• Increase reach of existing preventive interventions in the preconception period, especially family planning, and including adolescent-friendly services.

• Ensure that every woman receives the high-quality care she needs during pregnancy, birth and postnatally, especially if she is at risk of preterm birth. There should be greater emphasis on the universal provision of antenatal corticosteroids, building on the work of the UN Commission on Life-Saving Commodities for Women and Children as an opportunity to accelerate progress.

• Undertake immediate action to scale up KMC as a standard of care for all preterm babies under 2,000 grams, regardless of resource setting.

• Improve methods for diagnosing and treating prematurity-related impairment in childhood.

• Ensure that every family has the support they need, immediately after birth of a premature baby, following its loss, or living with a child with prematurity-associated disability.

Parents, advocates and civil society have captured the attention of governments and monitored progress in the United States through support of an annual Premature Birth Report Card. The Report Card, a familiar means of assessing progress for school-age children, has been a powerful tool used in the United States to prevent preterm birth and its serious health consequences. These grades, used as a rallying point, have helped bring visibility and promote change. Issued by the March of Dimes every year since 2008, the Report Cards assign a letter grade to the United States and to each of 50 state governments. In addition, they summarise the actions that must be taken to fund prevention programs, address health care access and bring about needed change in health care systems.

Transparency and objectivity of the data and analysis are important factors in the success of the Report Cards. Each year, great care is taken to explain the methodology for grade determination and the basis of comparison to other states. Use of the Report Card grades by state governments has grown since the Report Cards were first launched in 2008, and coverage by local media is consistently strong.

One southern U.S. state, with the second highest preterm birth rate in the country, has received an "F" on its Report Card every year since 2008. The failing grade mobilised state health officials in early 2012 to launch a statewide initiative with the goal of reducing rates. In this state and many others, media events featuring prominent public officials are held to announce Report Card grades or report on state progress to address preterm birth. The U.S. Surgeon General has also participated in media outreach to publicise the Report Cards and their recommended actions.

Sustained effort by healthcare leaders and advocates at all levels, inside and outside of government, has elevated the issue of preterm birth on the nation's health agenda, contributing to an announcement of new federal resources to test promising practices in February 2012. Soon after, the Association of State and Territorial Health Officials (ASTHO) joined with the March of Dimes to ask state health officials to pledge to reduce preterm birth rates in their states, and the pledge was incorporated into Report Cards. Top health officials in every state, along with Puerto Rico and the District of Columbia, signed the pledge.

As federal and state governments devote attention and resources to the problem, the Report Cards will continue to mobilise stakeholders and mark progress.

More information is available at http://www.marchofdimes.com/mission/prematurity-reportcard.aspx

#### Inform

Improve the data for preterm birth rates, mortality, impairment and their causes, with regular tracking of coverage, quality and equity gaps, as is done through Countdown to 2015 and linked to the work of the Commission for Information and Accountability using the data for action and accountability, including the establishment of national birth registrations.

#### Innovate

Conduct multi-country collaborative research on the:

• Etiology of preterm birth, advancing the understanding of strategies to prevent and treat maternal health conditions associated with preterm birth (e.g., pre-eclampsia and gestational diabetes) and improving identification of diagnostic markers and related screening tools.

• Implementation research to develop and deliver innovations to reach the poorest.

**Figure 9:**
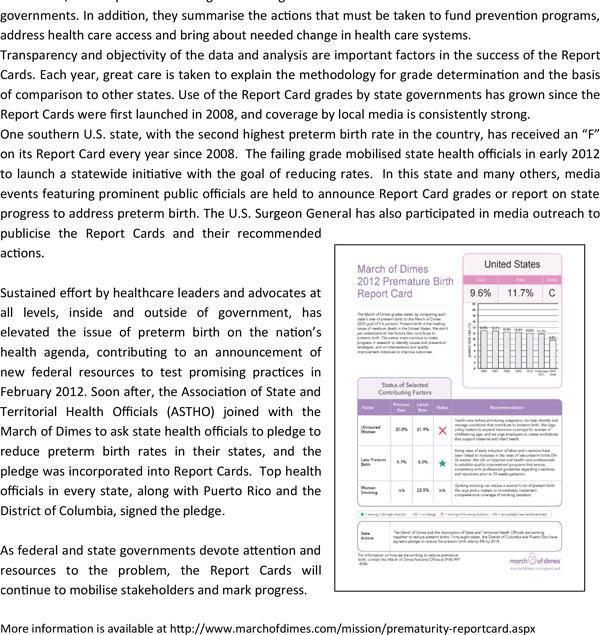
**Government - national integrated campaign for preterm births**.

**Figure 10:**
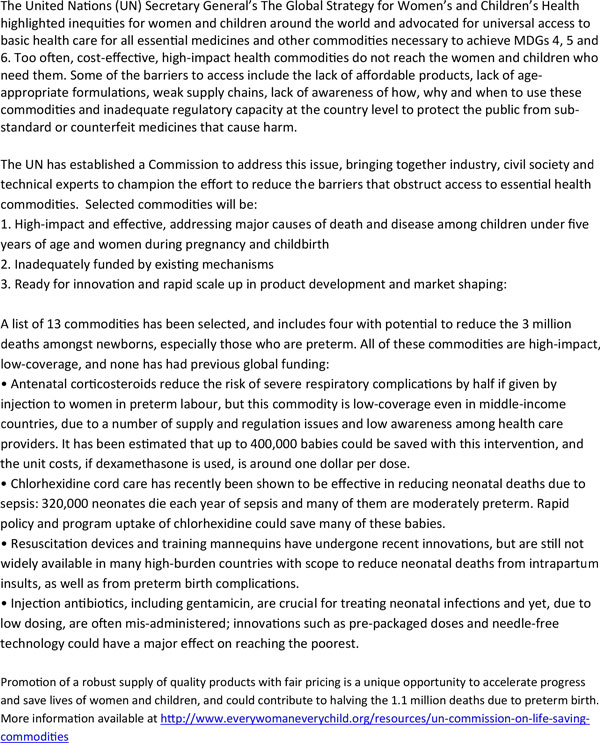
**The United Nations - Life-saving Commodities for Women and Children-- potential for action to reduce preterm deaths**.

**Figure 11:**
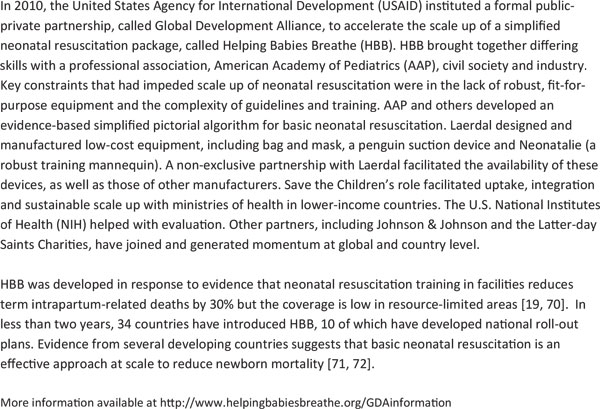
**Donors and philanthropic institutions - Helping Babies Breathe as an example of a public-private alliance to save newborns**.

**Figure 12:**
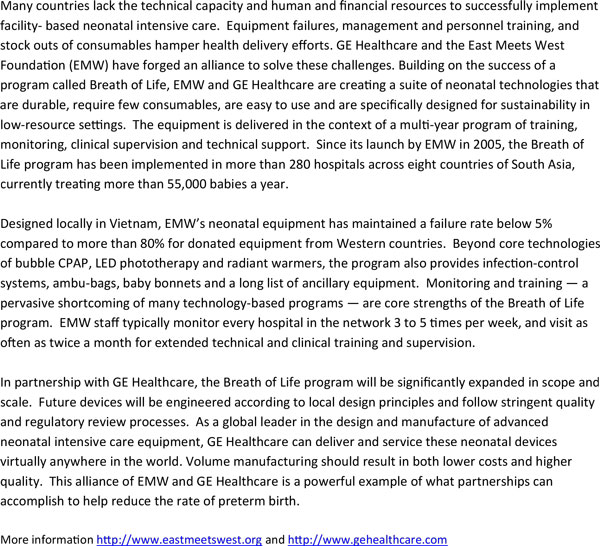
**The business community - Industry partnership for innovative technology for preterm baby care in Asia**.

**Figure 13:**
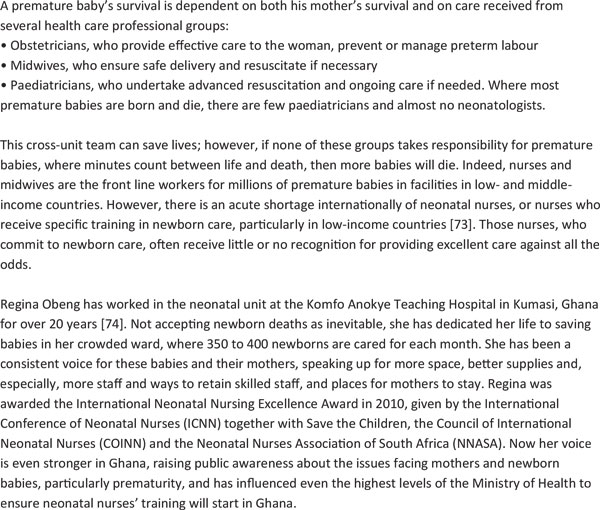
**Health care workers - Health care providers as champions of change for mothers and newborns**.

**Figure 14:**
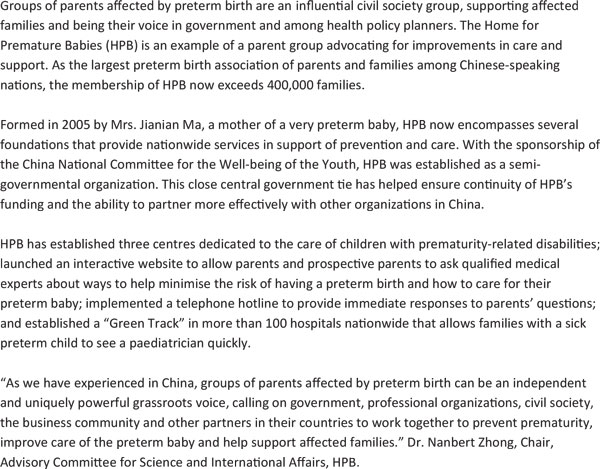
**Civil society - Chinese parents mobilising for their preterm babies**.

Table 3 details actions for the seven constituencies and Figures 9, 10, 11, 12, 13, 14 provide examples of action. This agenda is ambitious, yet it can and must be accomplished if the actions are to be given the visibility, funding and attention they deserve. To be successful in our goals, the constituencies identified must work together collaboratively and in partnership in ways that are transparent to all, vigorous and accountable.

**Table 3:** Everyone has a role to play: actions for the six key constituency groups involved in *Every Women Every Child*

**Governments and policy-makers at local, national, regional and global levels:** *Invest* • Set national targets for improved survival of premature babies and increase funding to ensuring equitable access to quality care to meet these targets by 2025. *Implement* • Strengthen health systems for quality maternal and neonatal care, including improved community awareness and demand for RMNCH services and adopt policies to promote universal access to quality preconception and maternal and perinatal services. *Innovate* • Promote the discovery, development and delivery of affordable and essential medicines, new technologies and novel models for training and services to prevent preterm birth and improve care of premature babies. *Inform* • Improve systems for collecting, evaluating and disseminating data on preterm birth rates, mortality, disability, quality of life and equitable coverage of evidence-based interventions to track progress towards MDGs 4 and 5 for maternal and child survival. **The United Nations and other multilateral organistions:** *Invest* • Support countries develop and align their national health plans, including costing and tracking implementation to achieve the health MDGs and preterm birth mortality-reduction targets. *Implement* • Define norms and guidelines to support efforts to improve women's and children's health, and encourage their adoption through provision of technical assistance and programmatic support for the prevention and treatment of preterm births. *Innovate* • Generate and disseminate evidence on preterm birth and provide a platform for sharing best practices, and use the UN Commodities Commission to address gaps for essential equipment and medicines (e.g., antenatal corticosteroids). *Inform* • Support the production, dissemination and use of coverage data for evidence-based interventions through the Countdown to 2015 and Commission for Information and Accountability through the independent Expert Review Group. **Donors and philanthropic institutions:** *Invest* • Provide sustained long-term support in line with national health policies and RMNCH plans that incorporate preterm births and are harmonised with other related global health initiatives. *Innovate* • Support high-priority research efforts to address solution gaps and implementation research to inform the scale up of evidence-based interventions to reduce preterm deaths. *Inform* • Promote transparent tracking of commitments and accountability and of long-term improvements in national health management and information systems. **The business community:** *Invest* • Invest additional resources to develop and adapt devices and commodities to prevent and treat preterm birth in low-income settings using innovative partnerships and business models. *Implement* • Scale up best practices and partner with the public sector to improve service delivery and infrastructure for prevention and management of preterm birth. *Innovate* • Develop affordable new diagnostics, medicines, technologies and other interventions, including social and behavioural change, for preterm birth and make them available to the most vulnerable and marginalised. *Inform* • Use and strengthen existing tracking systems for commodities and devices to improve supply chain logistics. **Academic and research institutions:** *Invest* • Agree upon and promote an innovative research agenda for prevention of preterm birth and improved pregnancy outcomes and implementation research to reduce deaths from preterm birth. *Implement* • Build capacity at research institutions, especially in low- and middle-income countries, and train professionals. *Innovate* • Advance policy development by improving the metrics for impairment outcomes as well as preterm birth rates, and link to other pregnancy outcomes, reporting on trends and emerging issues relating to preterm births. *Inform* • Strengthen global networks to disseminate new research findings and best practice related to preterm birth through leveraging the momentum from Born Too Soon and commitments of these institutions. **Health care workers and their professional organisations:** *Invest* • Advocate for and participate in evidence-based training, deployment and retention of workers with the necessary skills to address the burden of preterm birth. *Implement* • Use evidence-based standards to prevent or treat preterm births; implement training; and update curricula with evidence-based interventions. Treat women, newborns and children with respect and sensitivity. *Innovate* • Work in partnership to provide universal access to the essential package of interventions, including both prevention and care, and involving task shifting where appropriate. *Inform* • Improve data collection to track preterm births and measurements, such as consistent assessment of gestational age, birthweight, cause of death, data on impairment and retinopathy of prematurity. **Civil society:** *Invest* • Advocate for increased attention to the health of women, newborns and children through strengthening parent groups and conducting national campaigns focusing on preterm birth. *Implement* • Strengthen community and local capabilities to scale up implementation of interventions for preterm birth and support families who have lost babies or require long-term support for disability. *Innovate* • Develop and test innovative approaches to deliver essential services for prevention and care, particularly ones aimed at the most vulnerable and marginalised people. *Inform* • Educate, engage and mobilise communities to improve health education and care, beginning in adolescence; promote cost-effective solutions; track progress and hold all stakeholders at global, regional, national and local levels accountable for their commitments; promote accountability through the issuance of annual Countdown to 2015 country data profiles and global and national reports that document preterm birth rates and associated mortality and coverage of evidence-based interventions.

Source: adapted from *Born Too Soon *report, chapter 6 [48]

All of the partners, donors and contributors involved in the *Born Too Soon *movement see the report and this supplement as important next steps towards a world where every woman, every newborn and every child is given the best chance to survive and thrive.

## Conclusion - Together rapid change is possible

Over the last decade, the world has changed. Just as it is no longer acceptable for people with HIV/AIDS to remain untreated because they live in poor countries, it is no longer acceptable for women to die while giving birth. Likewise it should be unacceptable for almost 3 million newborns, to die, including those who are born too soon. Over three-quarters of premature babies who die could be saved if basic care reached them and their mothers. Rapid progress is possible. At the same time, research and innovation for preterm birth prevention is urgent. These actions would reduce disability and chronic disease, improve reproductive and maternal health, and build sustainable health systems. We need more frontline health workers who are skilled and confident in newborn care. We need facilities equipped with life-saving commodities, and girls, and women who are educated, and enabled, can protect their own health, and that of their babies.

## List of abbreviations used

KMC: Kangaroo Mother Care; PMNCH: Partnership for Maternal, Newborn and Child Health; pPROM: Prelabour premature rupture of membranes; RMNCH: Reproductive, Maternal, Newborn and Child Health; STI: Sexually Transmitted Infection; WHO: World Health Organization.

## Competing interets

The authors declare that they have no conflict of interest

## Authors' contribution

The chapter was coordinated by JEL with MVK; drafted by JEL, MVK, CPH, EL and all other authors reviewed and contributed.

## Funding

The time of JEL and MVK was funded by a grant from Bill & Melinda Gates Foundation to Save the Children's Saving Newborn Lives programme. The Born Too Soon report was funded by March of Dimes, the Partnership for Maternal, Newborn and Child Health and Save the Children. The Born Too Soon series in Reproductive Health was funded by March of Dimes and Save the Children. Funding from Save the Children is provided by a grant from Bill & Melinda Gates Foundation to Save the Children's Saving Newborn Lives programme.

## Supplementary Material

Additional file 1 **In line with the journal's open peer review policy, copies of the reviewer reports are included as additional file **1. Click here for file
