# Supplementary material for: Born Too Soon: Accelerating actions for prevention and care of 15 million newborns born too soon
Source: Reprod Health. 2013 Nov 15;10(Suppl 1):S6. doi: 10.1186/1742-4755-10-S1-S6 (PMC3828574; doi:10.1186/1742-4755-10-S1-S6)
Supplement: Additional file 1 — In line with the journal's open peer review policy, copies of the reviewer reports are included as additional file 1. [file 1742-4755-10-S1-S6-S1.doc]

**Referee’s comments to the authors– this sheet WILL be seen by the author(s) and published with the article**

| Title | Paper 6: Accelerating Action for Prevention and Care of 15 Million Newborns Born Too Soon |
| --- | --- |
| Author(s) | Joy E Lawn, Mary V Kinney, José M Belizan, Elizabeth Mary Mason, Lori McDougall, Jim Larson, Eve Lackritz, Ingrid Friberg & Christopher P Howson |
| Referee’s name | Cynthia Stanton |

**When assessing the work, please consider the following points, where applicable:**

1. Is the question posed by the authors new and well defined?
2. Are the methods appropriate and well described, and are sufficient details provided to replicate the work?
3. Are the data sound and well controlled?
4. Does the manuscript adhere to the relevant standards for reporting and data deposition?
5. Are the discussion and conclusions well balanced and adequately supported by the data?
6. Do the title and abstract accurately convey what has been found?
7. Is the writing acceptable?

Please make your report as constructive and detailed as possible in your comments so that authors have the opportunity to overcome any serious deficiencies that you find and please also divide your comments into the following categories:

- Major Compulsory Revisions (which the author must respond to before a decision on publication can be reached)
- Minor Essential Revisions (such as missing labels on figures, or the wrong use of a term, which the author can be trusted to correct)
- Discretionary Revisions (which are recommendations for improvement but which the author can choose to ignore)

Where possible please supply references to substantiate your comments.

When referring to the manuscript please provide specific page and paragraph citations where appropriate.

**General comments:**

It is great to see this issue gaining a higher profile and provides hope that the post 2015 MDG-agenda will continue to include a focus on issues related to birth outcomes The authors assembled for this paper constitute an impressive group with indepth and wide-ranging expertise re: preterm birth in high and low income countries.

I assume it is understood that I have not read the 5 papers that precede this article. However, I also assume that each paper should be able to stand on its own.

Major Compulsory Revisions

I have read this paper four times. It compiles a great deal of helpful information. However, the organization of the paper is such that I felt lost as I moved from paragraph to paragraph, and at times from sentence to sentence within paragraphs. A few key examples:

1) My understanding is that the objectives of the paper are to: summarize evidence-based interventions, the implications for integrating and scaling up currently available interventions and lives saved as a result of existing interventions.

Having finished the paper, it became clear that the paper is focused on low/middle income countries, though this is not stated in the title, abstract or Introduction.

If a main objective of the paper is to summarize evidence-based interventions, I do not see that Discovery science or Development of new interventions (included in Table 1) fits into these objectives. If a discussion of the implications of scale up is an objective, more serious and structured discussion of cost and cost-effectiveness is needed and a comparison of the

As a reader, I found Box 3 with suggestions a call to action specific to various constituencies very helpful.

**Major compulsory revisions:**

**Minor essential revisions:**

**Discretionary revisions:**

*(continue on the next sheet)*

*Continued*

Discovery science or Development of new interventions fits within these objectives.

The authors state that previous papers in the supplement identify gaps in coverage, quality, metrics and equity. If so, why are they covered in this paper? Furthermore, there are only 2 paragraphs on this series of complex subjects, each of which is treated in a cursory manner, so I am not convinced they need to be here.

If the objective is to summarize evidence-based interventions, why start the paper with an Action Framework? Don’t the readers need the basics first on what works? Then, the implications for scale up? Then, recommendations re: an Action framework? Again, the organization of paragraphs kept me confused about where the paper was going.

After multiple readings, it seems to me that though the authors state that this paper is within “the context of the wider health system”, this paper is really about health facility-based care. The few sentences (~2) referring to community-based care are mostly focused on efforts to get women to health facilities. Consequently, to help orient the reader, I would suggest stating up front that this paper focuses on health facility-based care (and interventions for women to access such care).

The first objective of the paper is to summarize effective interventions. Figures 1 and 2 are dense with highly summarized information which is hardly touched upon at all in the manuscript. Having read the Intro I would have expected this to be the main body of the paper. Figure 1 refers to ANC for women at risk of preterm birth, but nowhere is it mentioned what the risk factors are. The term PROM is mentioned in Figures and Tables but is not mentioned at all in the text of the paper. Antenatal corticosteroids are mentioned frequently in the text of the paper but nowhere is it described what they do or what problem they address (perhaps it is a given that everyone knows this, but I bet not; nor is there any discussion of the requirements of storing the drug, which cadre of provider is generally authorized to administer them, etc.).

Given that an objective of the paper is to discuss implications of scale up, I think it would be important/helpful to provide some information on cost-effectiveness of the “new high-impact cost-effective interventions currently at low coverage”, and to compare the cost-effectiveness of these to other birth-related interventions being promoted or in use.

Editorial comments:

- The info in the first sentence in the Abstract does not exist anywhere in the paper.
- There are references in the Abstract and the main body of the paper to reports such as Every Woman Every Child, or Born Too Soon – which assumes the reader knows what these are and I suspect many will not.
- On page 4, 5^th^ paragraph, it is stated: More than 60% of all premature babies are born in South Asia or SSAfrica….. The implication is that these world regions disproportionately contribute preterm births, but in fact about 55% of global births occur in these two regions (depending on whose numbers you use). So, yes, they have a bit more than their share, but really not many more.
- Page 10, 3^rd^ paragraph: I had a difficult time following the text here. I suggest the authors be specific when they are referring to a reduction in mortality rates and in numbers of deaths.
- Figure 4: the y axis is: Average annual rate change of preterm specific NMR (and NMR is not included in the title of the Figure). I assume NMR is not needed since the denominator would still be all births. In any case, the y axis and the title should be consistent.
- Table 3: the title refers to six key consistency groups – I assume this is constituency groups.

In summary, there is a great amount of important information in this paper, but the organization of the paper really hampered my ability to understand it. I hope my comments have been helpful and I look forward to the supplement hitting the printed/electronic page.

**Referee’s comments to the authors– this sheet WILL be seen by the author(s) and published with the article**

| Title | Paper 6: Accelerating Action for Prevention and Care of 15 Million Newborns Born Too Soon |
| --- | --- |
| Author(s) | Joy E Lawn, Mary V Kinney, José M Belizan, Elizabeth Mary Mason, Lori McDougall, Jim Larson, Eve Lackritz, Ingrid Friberg & Christopher P Howson |
| Referee’s name | Wally Carlo |

**When assessing the work, please consider the following points, where applicable:**

1. Is the question posed by the authors new and well defined?
2. Are the methods appropriate and well described, and are sufficient details provided to replicate the work?
3. Are the data sound and well controlled?
4. Does the manuscript adhere to the relevant standards for reporting and data deposition?
5. Are the discussion and conclusions well balanced and adequately supported by the data?
6. Do the title and abstract accurately convey what has been found?
7. Is the writing acceptable?

Please make your report as constructive and detailed as possible in your comments so that authors have the opportunity to overcome any serious deficiencies that you find and please also divide your comments into the following categories:

- Major Compulsory Revisions (which the author must respond to before a decision on publication can be reached)
- Minor Essential Revisions (such as missing labels on figures, or the wrong use of a term, which the author can be trusted to correct)
- Discretionary Revisions (which are recommendations for improvement but which the author can choose to ignore)

Where possible please supply references to substantiate your comments.

When referring to the manuscript please provide specific page and paragraph citations where appropriate.

**General comments:**

This is an excellent overview of neonatal mortality secondary to prematurity. Most aspects of preterm labor and preterm baby care are addressed but it is difficult to compare the effectiveness of the various interventions. A comprehensive continuum of care is presented at the expense of not distinguishing the most effective interventions from those not likely to be effective, particularly in LMIC. For example, marked emphasis is placed on prevention of prematurity although this intervention has been minimally effective in reducing mortality from prematurity even in high income countries.

**Major compulsory revisions:**

Page 3, second paragraph: The case for research to prevent prematurity is overstated. It is stated that “more research is urgently needed....” The research to prevent prematurity in the US and European countries has not resulted in a reduction in prematurity rates other than late preterm births. Indeed, prematurity rates have been on the rise other than the recent decrease in late preterm babies. This decrease in late preterm births is unlikely to result in a major reduction in neonatal mortality as their mortality rate is very low.

*(continue on the next sheet)*

*Continued*:

In contrast, for preterm infant care there is no call for research. Preterm care research specifically for developing countries is necessary. Innovative low cost and effective interventions are needed as these countries cannot afford the expensive NICU care of the West. Scaling up of some interventions is appropriate but others need to be evaluated. This paragraph and the following one should be revised accordingly.

Page 4: The paragraph on antenatal corticosteroids does not address the most important benefits of this intervention. The paragraph addresses only prevention of RDS but does not address reduction in mortality and reduction in long term morbidities.

Page 4, 4^th^ paragraph: It is stated that “care of premature babies is primarily an action gap.” However, most proven interventions have not been tested in resource-limited clinical settings. Even KMC which appears to be one of the most effective interventions in reducing mortality is LMIC has resulted in a relatively small (1% in the LBW meta-analysis by Conde-Agudelo) absolute risk reduction in mortality. A larger effect may be expected in LMIC but it is unclear on what basis such a large effect in mortality has been suggested by some authors). In discussing the results of their recent very large KMC trial, Sloan et al stated, “It is inappropriate to implement community kangaroo mother care at this time on the presumption of benefit or the assumption of no harm.” Further research is needed to optimize KMC. For example, in research studies as many as 50% of infants develop hypothermia despite KMC.

Page 4, last paragraph: It is an overstatement that almost all deaths of infants >32 weeks of gestation can be prevented with ENC. The effectiveness of some of the interventions addressed may not have been as effective in reducing mortality as stated in this paper.

**Minor essential revisions:**

Prenatal care may have limited effects on prematurity rates as addressed by the authors when tocolytics are mentioned. For example, the rates of preterm birth secondary to pre-eclampsia and maternal hypertension have not decreased with increased care. Identification of these women is important for their care as well as for antenatal corticosteroids but increased pre-eclampsia/maternal hypertension care may lead to more (not less) prematurity (but also more mothers’ lives saved).

Page 8, Building the Platform paragraph: Too much emphasis is placed on interventions that have not been effective in developed countries. For example, the sentence “multi-country studies teaching pregnant women with improved and accurate gestational dating...” is not supported by strong evidence of effectiveness in reducing maternal or neonatal mortality.

**Discretionary revisions:**

Page 5, paragraph 3: A continuum of care and integrated services delivery packages appear to be ideal but do not distinguish interventions by their likely effectiveness in reducing mortality. Resuscitation is presented side by side with CPAP but the latter has not been tested in trials in developing countries. It is stated that these interventions are supported by systematic review but it is important to focus on data from LMIC when available.

Family planning is mentioned as a way to reduce death from prematurity but family planning is readily available in high-income countries and has not resulted in decreased prematurity rates.
